# Enhancement of dose distribution using tissue compensators in boron neutron capture therapy

**DOI:** 10.1002/acm2.70392

**Published:** 2025-11-21

**Authors:** Akinori Sasaki, Naonori Hu, Mai Nojiri, Kazuhiko Akita, Syuushi Yoshikawa, Yasushi Kohigashi, Keiji Nihei, Teruhito Aihara, Satoshi Takeno, Yuki Yoshino, Hiroki Tanaka, Koji Ono

**Affiliations:** ^1^ Kansai BNCT Medical Center Osaka Medical and Pharmaceutical University Osaka Japan; ^2^ Institute for Integrated Radiation and Nuclear Science Kyoto University Kyoto Japan; ^3^ BNCT Joint Clinical Institute Osaka Medical and Pharmaceutical University Osaka Japan; ^4^ Department of Radiation Oncology Osaka Medical and Pharmaceutical University Hospital Osaka Japan; ^5^ Department of Otorhinolaryngology Head and Neck Surgery Osaka Medical and Pharmaceutical University Hospital Osaka Japan; ^6^ Department of Radiology Kyoto Prefectural University of Medicine Kyoto Japan

**Keywords:** BNCT, monte carlo simulation, neutron scatter, tissue compensator

## Abstract

In BNCT for head and neck tumors delivered with a horizontal beam, the presence of missing tissue near the beam path can cause reduction in neutron fluence rate in adjacent tissue regions due to the loss of scattered neutrons. These regions are referred to as neutron depleted areas. By placing a tissue equivalent compensator in these missing tissue regions, the lateral scatter equilibrium is partially restored, increasing the local neutron fluence rate and, consequently, the dose to the target volume while maintaining normal tissue exposure within tolerance limits.

## 1 INTRODUCTION

Boron neutron capture therapy (BNCT) is a form of radiotherapy that utilizes charged particles produced by the nuclear reaction between ^10^B and thermal neutrons.^1^ Because the resulting charged particles have a short range and a high linear energy transfer (LET), BNCT can selectively destroy tumor cells while minimizing damage to normal cells, provided that boron accumulates preferentially in the tumor cell.

In Japan, BNCT has been covered by the national health insurance system since June 2020 for patients with “unresectable locally advanced or locally recurrent head and neck cancer.” At our center, a total of 431 treatments were performed over a four‐year period from 2020 to 2024.

The NeuCure BNCT irradiation system is equipped with a horizontal beam port that allows treatment to be performed with the patient in either a seated or supine position. In the NeuCure BNCT system, a cyclotron accelerator is used to accelerate protons to approximately 30 MeV.^2^, ^3^ When the accelerated protons collide with a beryllium target, fast neutrons are generated. The fast neutrons generated at the target pass through the beam shaping assembly while undergoing repeated scattering. As a result, the neutrons extracted from the collimator have both continuous energy and an angular distribution.

To reduce unnecessary exposure and to ensure that an adequate neutron flux reaches the treatment site, it is essential in BNCT patient positioning to place the target area as close as possible to the collimator.

In BNCT, the treatment is usually delivered in a single fraction with a single irradiation field, and the treatment time typically ranges from 30 to 60 min. For seated treatments, patients must maintain a position different from their natural resting posture for an extended period, which can be uncomfortable. In contrast, supine positioning allows for a more comfortable posture, but in some cases, it is difficult to bring the treatment site close enough to the collimator due to interference from the shoulders.

At our center, the development of an extended collimator has made it possible to perform supine treatments without discomfort, while avoiding shoulder interference and still positioning the treatment site sufficiently close to the collimator.^4^ Since the introduction of the extended collimator in March 2022, nearly all treatments at our facility have been performed in the supine position using this device.

When performing irradiation in the supine position using a horizontal beam, regions of reduced neutron fluence, referred to here as neutron‐depleted regions, may form within the irradiation field depending on the location of the target and the beam's position and angle. In the patient's anatomy, air gaps or missing tissue along the beam path can lead to a loss of scattered neutrons that would otherwise contribute to the local neutron field. Since neutrons scatter not only in depth but also laterally, the absence of surrounding scattering material reduces the thermal neutron flux in adjacent tissues. By placing a tissue equivalent object (tissue compensator) in the missing tissue area, neutron scattering within the compensator can be restored, increasing the number of neutrons that scatter back into the target volume. In treatments using x‐rays or electron beams, tissue compensators are commonly employed with the aim of achieving a uniform dose distribution.^5^, ^6^ In our practice, a tissue‐equivalent bolus^7^ commonly used in x‐ray or electron beam radiotherapy, was used as a compensator to improve the dose distribution in BNCT treatment planning. Specifically, the Clearfit bolus (Fujidenoro Co., Ltd.) with a density of 1.02 g/cm^3^ was used. The elemental composition ratio provided by the manufacturer is H:C:N:O = 10.26:5.27:0.04:1.61. Further information on the application of this bolus in a BNCT irradiation field are available elsewhere.^7^

In this report, we present a representative case from our center in which a tissue compensator was used in BNCT. We also describe the cases in which a tissue compensator was employed between 2020 and 2024, focusing on the enhancement in dose distribution to the tumor.

## 2 CASE PRESENTATION

**FIGURE 1:**
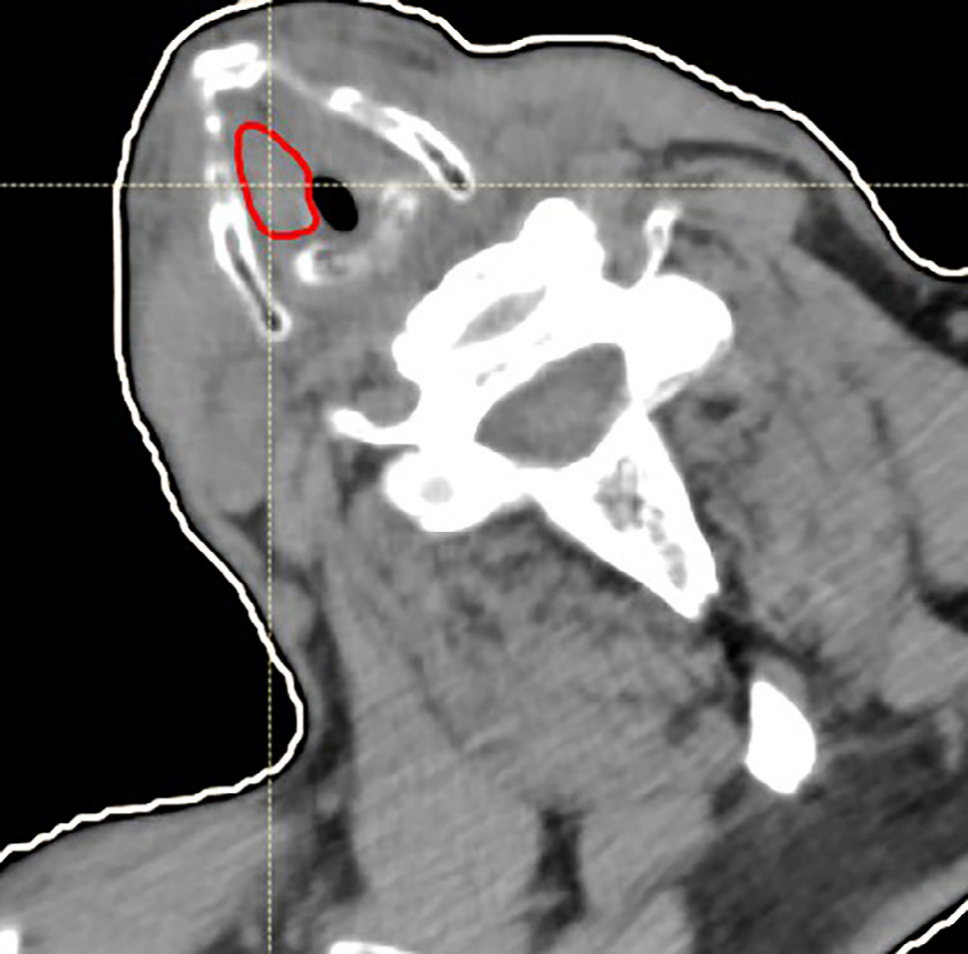
Axial CT image of the patient treated with BNCT. The GTV location is outlined in red.

We report a case in which BNCT with a tissue compensator was used at our center for laryngeal cancer of the right glottis. The gross tumor volume (GTV) was defined at the location indicated in Figure 1. The patient modelling and treatment planning process was performed using RayStation treatment planning system (TPS). The dose calculation was performed using the NeuCure dose engine.^8^

**FIGURE 2:**
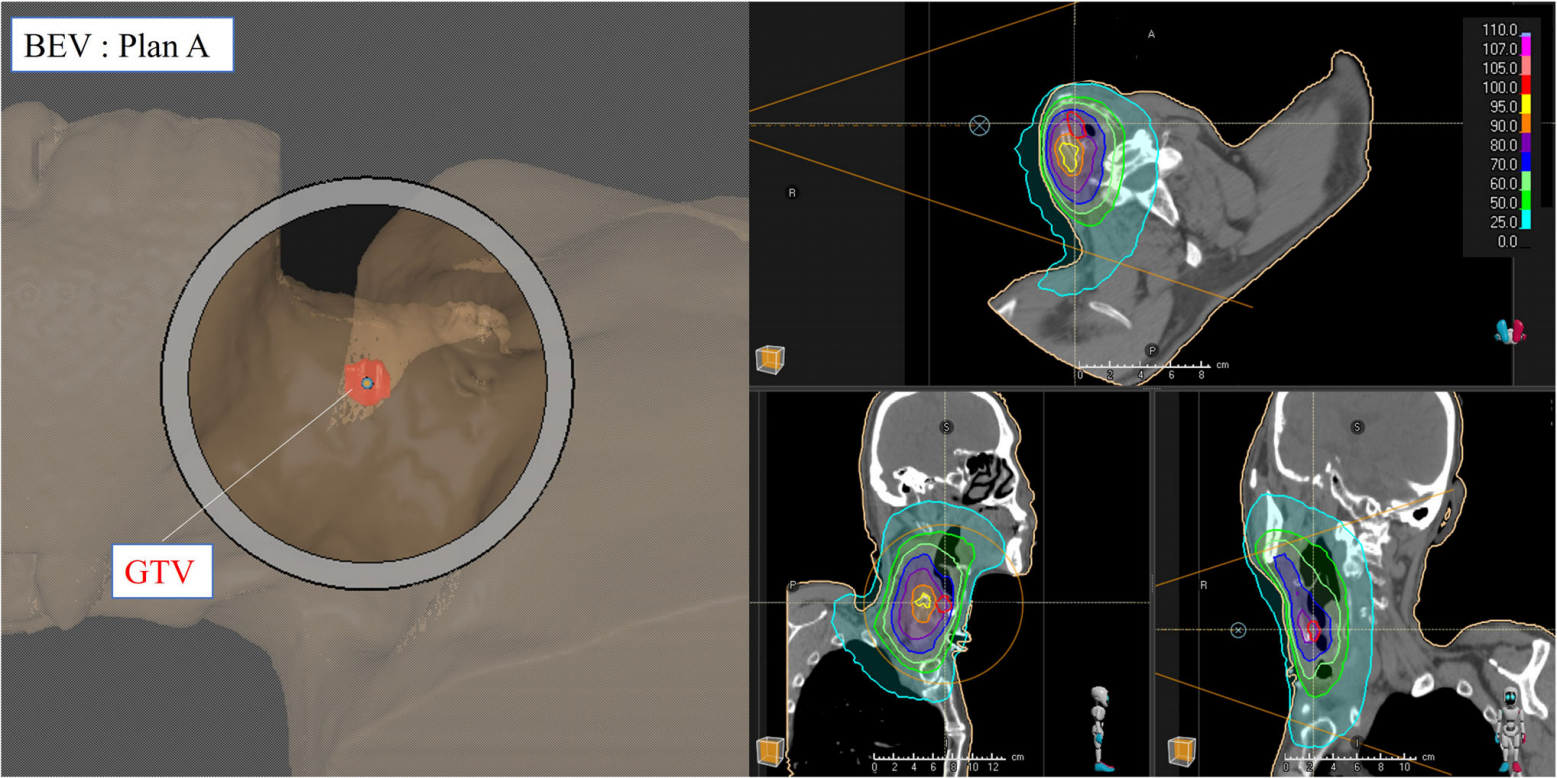
Screen capture of the treatment plan of irradiation plan A generated using RayStation. Left) BEV, Right) thermal neutron distribution.

In radiation therapy, it is common for the beam axis to pass through the target region (i.e. the beam isocenter inside the GTV). An example of such beam arrangement, referred to as plan A, is shown in Figure 2. The image shows the beams eye view (BEV) of plan A and the corresponding thermal neutron distribution.

**FIGURE 3:**
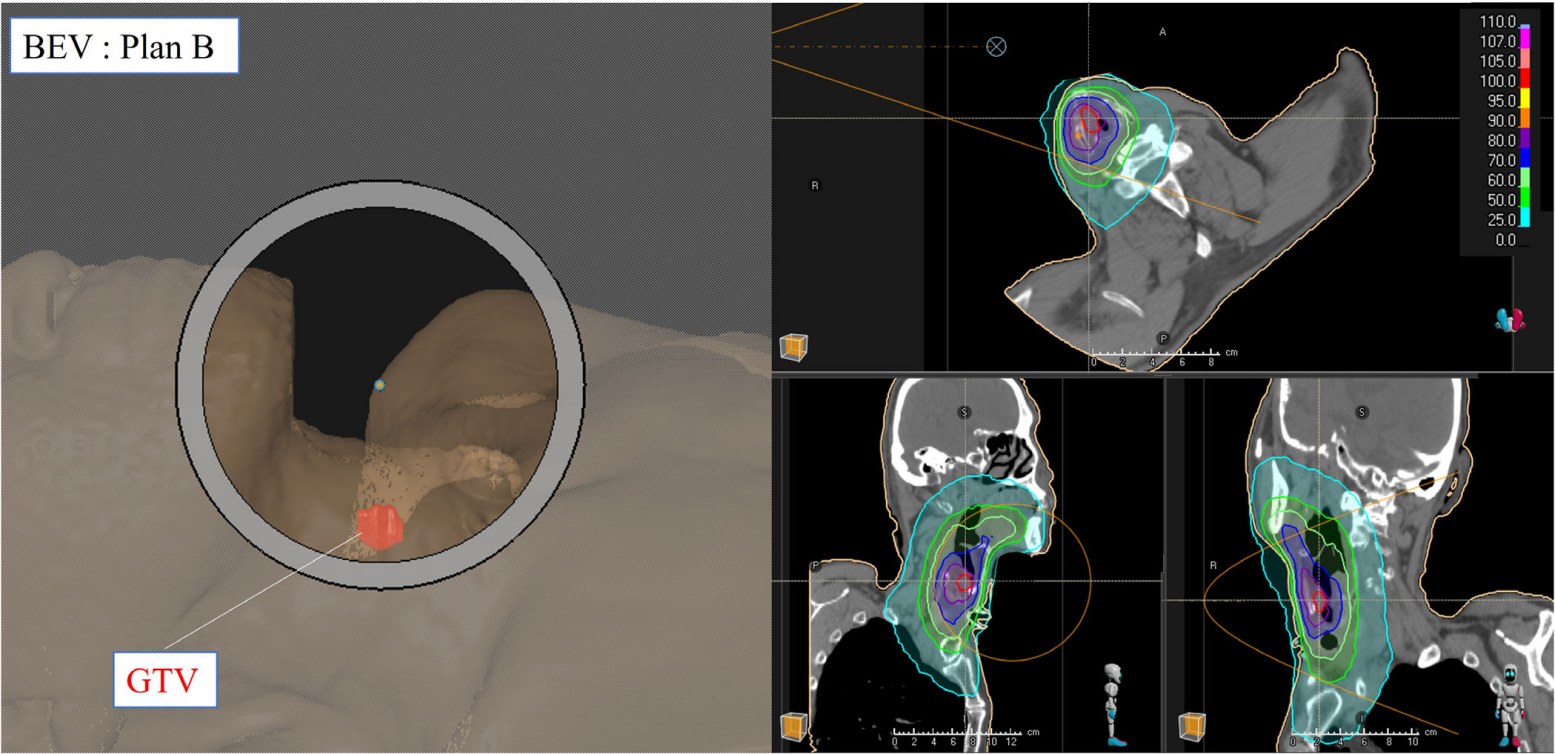
Screen capture of the treatment plan of irradiation plan B generated using RayStation. Left) BEV, Right) thermal neutron distribution.

Here, the thermal neutron distribution shows that the peak thermal neutron flux is located dorsal to the GTV. Therefore, to align the peak thermal neutron flux inside the GTV, it is necessary to shift beam isocenter (anteriorly). This plan is referred to as plan B and the corresponding thermal neutron distribution is shown Figure 3.

By shifting the beam isocenter anteriorly, the peak thermal neutron flux is brought closer to the GTV compared with plan A, indicating an improvement in the dose within the GTV. However, as can be seen on the BEV image, a large air gap exists within the field where neutrons pass through without interaction, resulting in a significant number of “wasted” neutrons. Not only will this increase the irradiation time, but it has the potential to increase the out‐of‐field dose of the patient.

**FIGURE 4:**
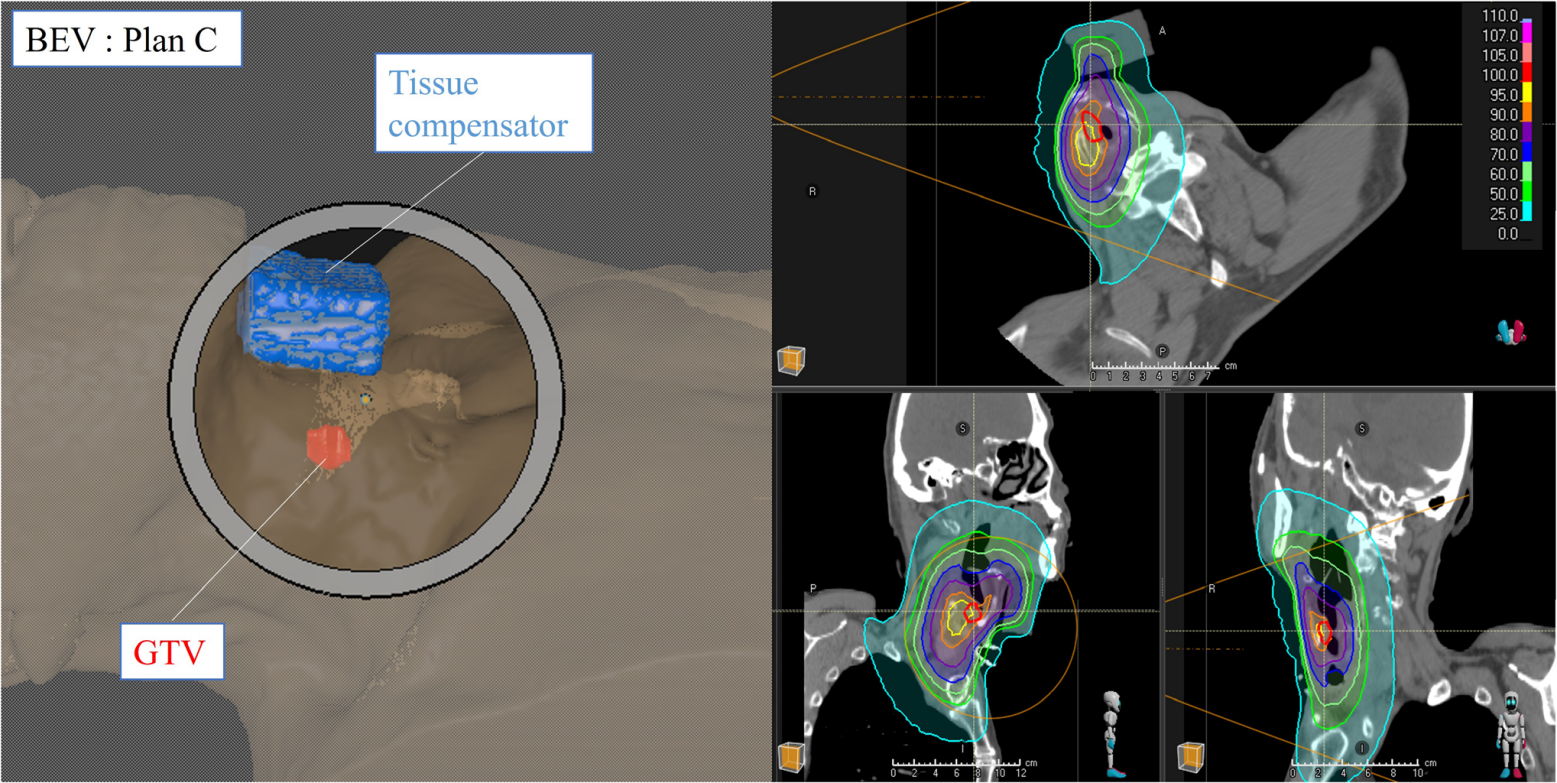
Screen capture of the treatment plan of irradiation plan C generated using RayStation. Left) BEV, Right) thermal neutron distribution.

The BEV and thermal neutron distribution with a tissue compensator placed in this space is shown in Figure 4 (plan C). A 3 cm thick tissue equivalent bolus material was placed in front of the throat region of the patient. By placing the tissue compensator on the patient, the peak thermal neutron flux coincides with the position of the GTV, indicating an improved thermal neutron flux distribution.

### 2.1 Dose calculation and comparison

**FIGURE 5:**
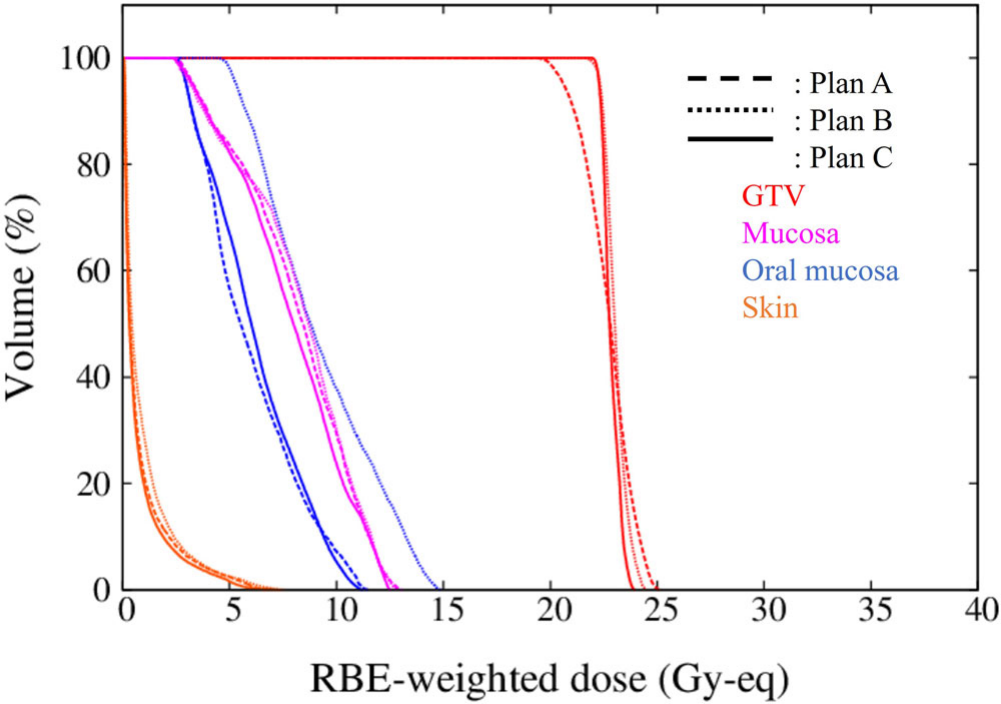
DVH of irradiation plan A, B, and C.

Dose–volume histograms (DVHs) for each plan are shown in Figure 5. All plans are prescribed to achieve D5% ≤ 12 Gy‐eq to the mucosa.

The use of the tissue compensator resulted in a significant improvement in the dose to the GTV when compared with plan A. The irradiation times for plan A, B, and C were 31.0 min, 43.8 min, and 30.3 min, respectively.

The homogeneity index (HI) for the GTV was defined as follows^9^:

$$\mathrm{HI}=\frac{D_{2\%}-D_{98\%}}{D_{50\%}}$$

**FIGURE 6:**
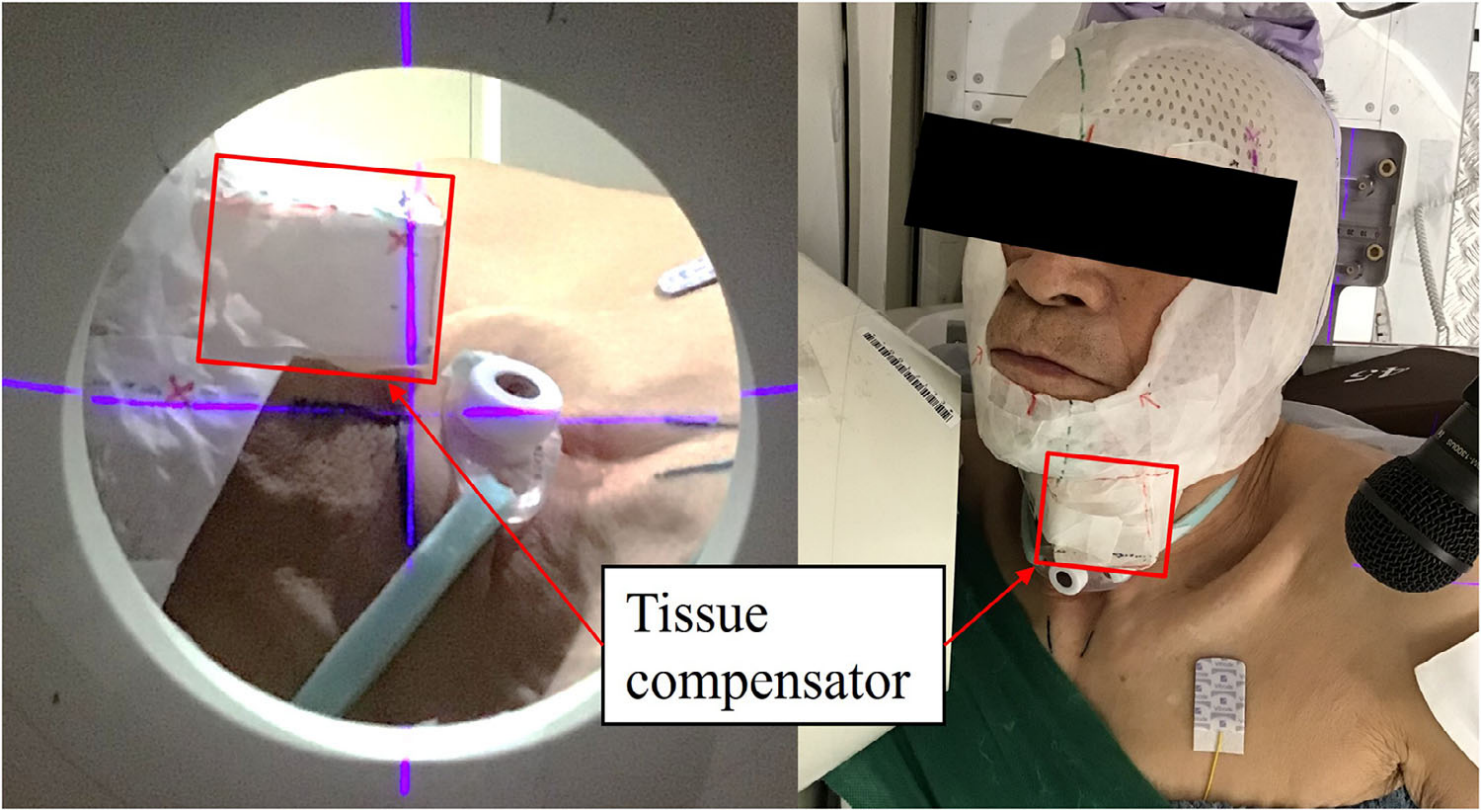
Image of the treatment with the tissue compensator in place.

The calculated HI values for each plan were $HI_{A}$​ = 0.20, $HI_{B}$​ = 0.09, and $HI_{C}$​ = 0.07. The use of a tissue compensator improved the HI. An actual photo of the patient treatment showing the tissue compensator is shown in Figure 6.

### 2.2 Summary of all cases treated with tissue compensators

**FIGURE 7:**
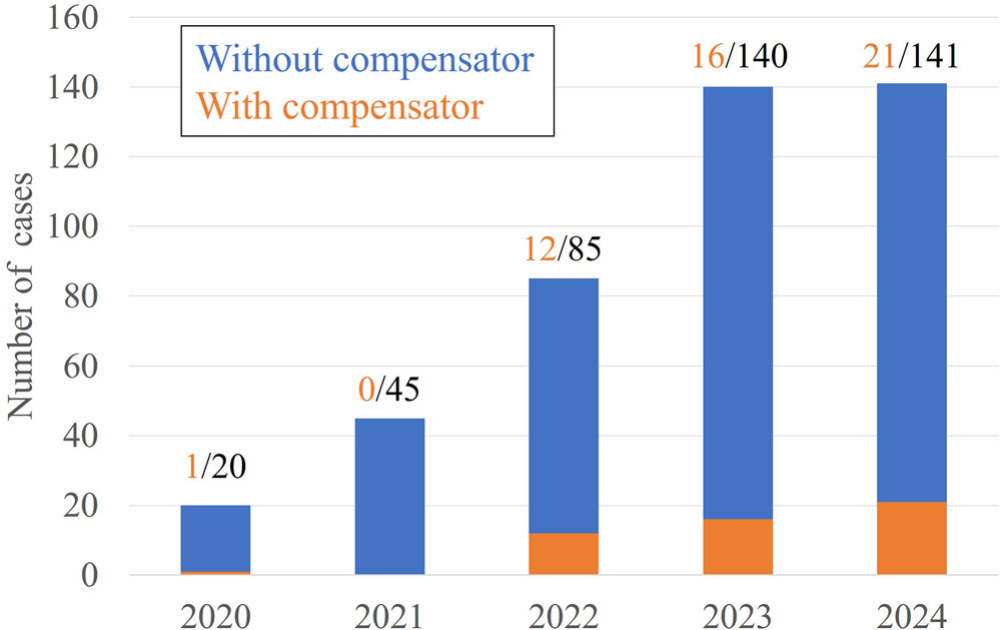
Number of cases by fiscal year.

At our center, a total of 431 BNCT treatments were performed from 2020 to 2024. The number of cases per year and the number of cases in which a tissue compensator was used is shown in Figure 7.

**FIGURE 8:**
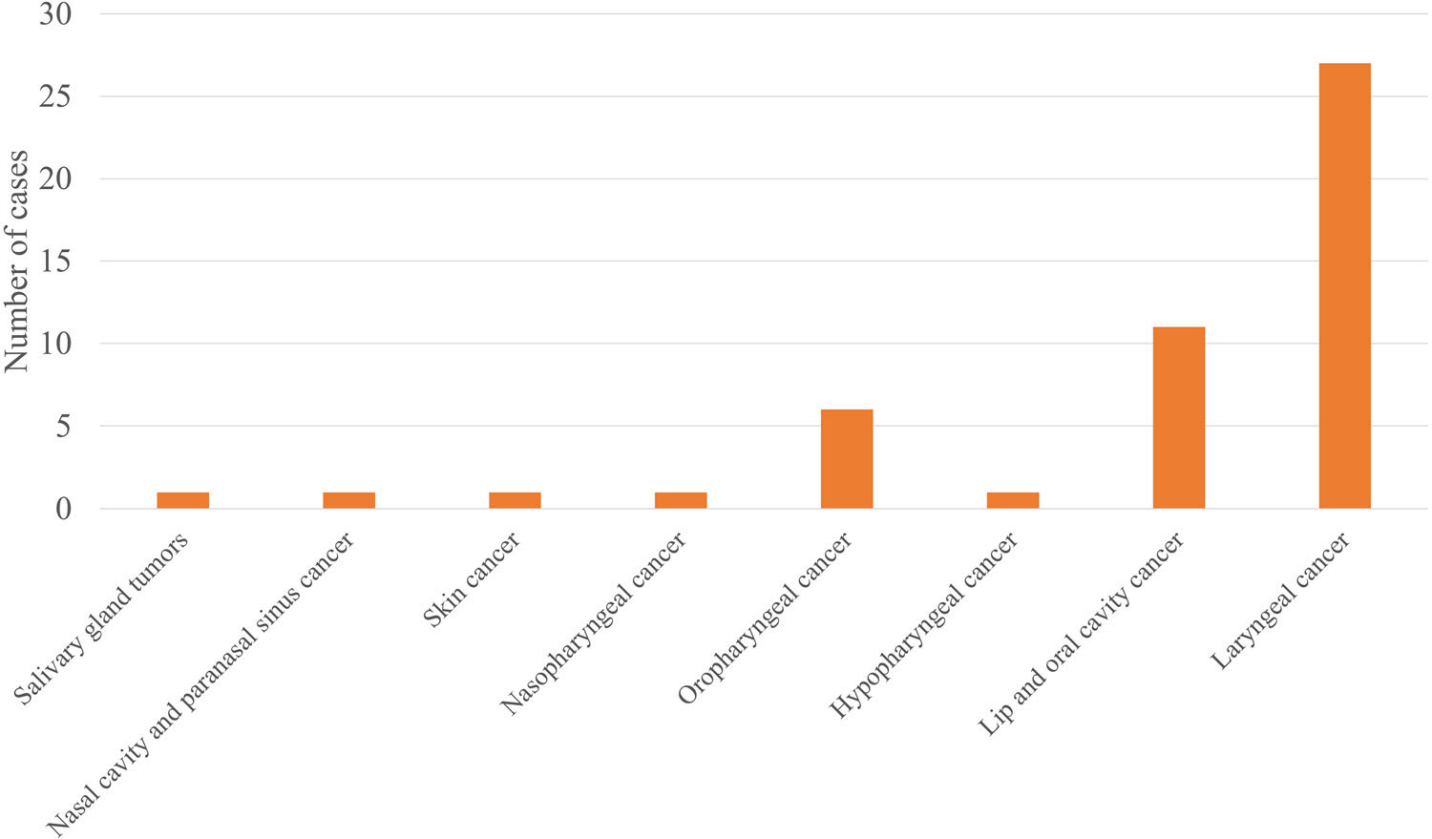
Number of cases using tissue compensators by site.

Out of the 431 total cases, tissue compensators were employed in 50 cases. It is important to note that these 50 cases involved the exclusive use of tissue compensators; cases utilizing a bolus for surface dose enhancement or for shielding organs at risk were excluded. For superficial target regions, dose distribution can sometimes be further optimized using a bolus to enhance surface dose.^10^ Additionally, shielding neutrons incident on normal tissues may further improve dose distribution within the target area. Nevertheless, since the objective of this study was to evaluate the dose improvement achieved specifically by the compensator, only cases in which the compensator was used alone were analyzed. The number of cases using tissue compensators by anatomical site is shown in Figure 8.

**FIGURE 9:**
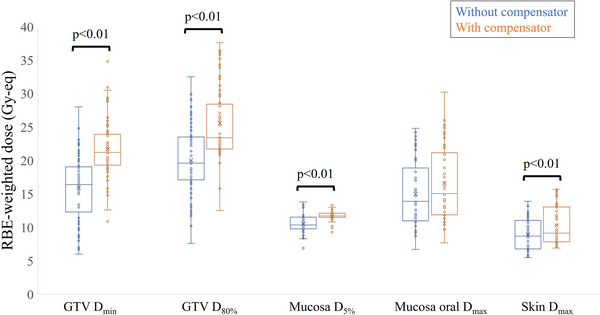
Statistical comparison of dose with and without a compensator for all cases.

Tissue compensators were primarily employed for treating lesions located in the larynx or oral cavity. In 50 cases that employed tissue compensators, we simulated identical treatment plans without the tissue compensator (by substituting the tissue compensator material with air) and compared the two plans. The comparison focused on the minimum dose to the GTV (*D*_min_) and the dose delivered to 80% of the GTV (*D*_80%_), as well as doses to representative organs at risk or surrounding tissues. As illustrated in Figure 9, the use of the tissue compensator significantly improved the dose to the GTV, while the dose to OARs, such as the mucosa, showed a slight increase.

## 3 DISCUSSION

This report describes the use of tissue compensators, a method to improve neutron distribution in BNCT. In the representative case, a comparison was made among irradiation plan A, B, and C. Compared with irradiation plan A, plan C showed almost no change in irradiation time while improving the *D*_min_, *D*_80%_, and the dose uniformity of the GTV. Compared with irradiation plan B, plan C was able to deliver an equivalent dose to the GTV in a shorter irradiation time and substantially reduced the dose to the oral mucosa. These results indicate that the use of a tissue compensator was an effective approach in this case.

Approximately 10% of all patients treated at our center utilized tissue compensators. Since the introduction of the extended collimator in March 2022, majority of irradiations have been performed in the supine position, resulting in an increase in the cases using tissue compensators. In 2024, tissue compensators were used in 21 out of 141 cases, and this number is expected to increase. This is because BNCT has shown favorable outcomes in the treatment of laryngeal cancer.^11^, ^12^ As BNCT enables the possibility of cure while preserving anatomical structures, it has been reported to be an effective treatment option for patients with laryngeal cancer who wish to retain their voice. Therefore, the use of the tissue compensator in BNCT for laryngeal cancer is expected to increase in the future.

At our center, one of the eligibility criterion for determining the suitability of BNCT is *D*_80%_ of the GTV exceeding 20 Gy‐eq.^11^ Among the 50 patients that used the tissue compensators, 49 met this criterion. Furthermore, among these, 34 cases would not have achieved this criterion without the use of the tissue compensator. The use of tissue compensators thus enables treatment for cases that would otherwise have been considered unsuitable for BNCT. On the other hand, doses to the mucosa, oral mucosa, and skin tended to increase with the use of the tissue compensator. Among these, dose increases to the mucosa and skin were statistically significant. The average dose to the mucosa, oral mucosa, and skin increased by 11.8%, 11.2%, and 16.6%, respectively. However, BNCT prescriptions are based on normal tissue tolerance doses. At our center, the tolerable normal tissue doses are set at 15, 12, 9, and 5 Gy‐Eq for the skin, pharyngeal mucosa, brain, and eyes, respectively.^11^ All normal tissue doses shown in the figure remain below tolerance limits. On the other hand, the average dose improvement for the tumor was 32.4% for *D*_80%_ and 48.4% for *D*_min_, respectively_._ Therefore, the use of tissue compensator improves GTV dose while maintaining normal tissue doses within tolerable levels.

When treating tumors located in the laryngeal region, the use of a tissue compensator proved effective in enhancing both the tumor dose and the dose homogeneity. Comparable improvements were also observed in the treatment of tumors within the oral cavity. When targeting the larynx without using tissue compensators, irradiation from the frontal direction is often considered. At our center, where the beam angle is fixed horizontally, frontal irradiation requires patients to be in a lateral decubitus position on the treatment couch. Since BNCT irradiation sessions can last up to 60 min, prolonged lateral positioning can cause discomfort for patients. Although irradiation in the seated position is possible, there are challenges related to long‐term immobilization and acquisition of planning CT images in the seated position, increasing the total set up uncertainty. The use of tissue compensators improves the dose distribution when treatment is performed in the supine position. In this study, we introduced a simple approach utilizing bolus material as a tissue compensator in BNCT. In the future, the application of more advanced techniques, such as 3D printing to fabricate precisely shaped compensators, may further enhance the treatment quality.

## 4 CONCLUSION

This report describes an irradiation technique using tissue compensators in BNCT and its effectiveness. The use of tissue compensators allows for improvement of tumor dose and homogeneity while keeping normal tissue doses within tolerable limits. With an expected increase in patient numbers, the use of tissue compensators is anticipated to expand the indication of BNCT.

## AUTHOR CONTRIBUTIONS

Conceptualization and methodology, N.H. Data analysis and process, A.S, M.N, N,H. Writing, A.S and N.H. Resources, K.A, S.Y, Y.K, T.A, S.T, Y.Y. Supervision, H.T, K.N, K.O. All authors have read and agreed to the published version of the manuscript.

## CONFLICT OF INTEREST STATEMENT

The authors declare no conflicts of interest.

## ETHICS STATEMENT

This study was approved by our institutional review board (approval no. 2021‐172‐1) and was conducted in accordance with the ethical standards of the 1964 Declaration of Helsinki.

## Data Availability

Research data are stored in an institutional repository and will be shared upon request to the corresponding author.
